# A novel cognitive behaviour therapy for bipolar disorders (Think Effectively About Mood Swings or TEAMS): study protocol for a randomized controlled trial

**DOI:** 10.1186/1745-6215-15-405

**Published:** 2014-10-24

**Authors:** Warren Mansell, Sara Tai, Alexandra Clark, Savas Akgonul, Graham Dunn, Linda Davies, Heather Law, Richard Morriss, Neil Tinning, Anthony P Morrison

**Affiliations:** School of Psychological Sciences, University of Manchester, Oxford Road, Manchester, M13 9PL UK; The Psychosis Research Unit, GMW Mental Health NHS Foundation Trust, Harrop House, Bury New Road, Prestwich, Manchester, M25 3BL UK; Centre for Biostatistics, Institute of Population Health, Jean McFarlane Building (1st Floor), Oxford Road, Manchester, M13 9PL UK; Centre for Health Economics, Institute of Population Health, Jean McFarlane Building (1st Floor), Oxford Road, Manchester, M13 9PL UK; Institute of Mental Health, School of Medicine, University of Nottingham, Wollaton Road, NG8 1BB Nottingham, UK; Bipolar UK, 11 Belgrave Road, London, SW1V 1RB UK

**Keywords:** Cognitive behavioral therapy, Controlled trial, Bipolar disorder, Recovery

## Abstract

**Background:**

Existing psychological therapies for bipolar disorders have been found to have mixed results, with a consensus that they provide a significant, but modest, effect on clinical outcomes. Typically, these approaches have focused on promoting strategies to prevent future relapse. An alternative treatment approach, termed ‘Think Effectively About Mood Swings’ (TEAMS) addresses current symptoms, including subclinical hypomania, depression and anxiety, and promotes long-term recovery. Following the publication of a theoretical model, a range of research studies testing the model and a case series have demonstrated positive results. The current study reports the protocol of a feasibility randomized controlled trial to inform a future multi-centre trial.

**Methods/Design:**

A target number of 84 patients with a diagnosis of bipolar I or II disorder, or bipolar disorder not-otherwise-specified are screened, allocated to a baseline assessment and randomized to either 16 sessions of TEAMS therapy plus treatment-as-usual (TAU) or TAU. Patients complete self-report inventories of depression, anxiety, recovery status and bipolar cognitions targeted by TEAMS. Assessments of diagnosis, bipolar symptoms, medication, access to services and quality of life are conducted by assessors blind to treatment condition at 3, 6, 12 and 18 months post-randomization. The main aim is to evaluate recruitment and retention of participants into both arms of the study, as well as adherence to therapy, to determine feasibility and acceptability. It is predicted that TEAMS plus TAU will reduce self-reported depression in comparison to TAU alone at six months post-randomization. The secondary hypotheses are that TEAMS will reduce the severity of hypomanic symptoms and anxiety, reduce bipolar cognitions, improve social functioning and promote recovery compared to TAU alone at post-treatment and follow-up. The study also incorporates semi-structured interviews about the experiences of previous treatment and the experience of TEAMS therapy that will be subject to qualitative analyses to inform future developments of the approach.

**Discussion:**

The design will provide preliminary evidence of efficacy, feasibility, acceptability, uptake, attrition and barriers to treatment to design a definitive trial of this novel intervention compared to treatment as usual.

**Trial registration:**

This trial was registered with Current Controlled Trials (ISRCTN83928726) on registered 25 July 2014.

## Background

Bipolar disorders are characterized by periods of depression and periods of elated or irritable mood (mania or hypomania), with around 2 to 6% of the general population meeting the criteria for diagnosis [1, 2]. The cost of bipolar disorders annually in the UK is £5.2 billion, projected to rise to £8.2 billion by 2026. Despite treatment, people with bipolar disorders remain depressed for the majority of time when not experiencing an episode. After apparent recovery from a bipolar episode, subsyndromal symptoms of depression remain in the long term, estimated at 30 to 50% of subsequent weeks [3, 4]. Depressive symptoms form the largest contribution to impaired functioning in bipolar disorder [5, 6]. At present, antidepressant treatment is relatively ineffective for these individuals [7].

There is a clear need for a more effective, cost-efficient intervention such as CBT (cognitive behavioral therapy). A number of reviews and meta-analyses of existing psychological interventions for bipolar disorders demonstrate mixed outcomes; yet overall, there are significant but modest effects [8–10]. A similar mixed set of outcomes were found in the most recently published trials, with most participants continuing to experience significant sub-clinical symptoms and disruption to life-functioning after receiving an intervention [11–13]. Thus, there remains a need for an alternative approach to managing bipolar disorders. Our group has been developing and piloting a case series for a new CBT approach known as TEAMS (Think Effectively About Mood Swings) based on an empirically supported model [14]. It involves a new integrative treatment approach that has the potential to benefit those people whose symptoms of depression, hypomania and anxiety have otherwise remained treatment-resistant and/or those with reduced functioning.

Our approach specifically targets current problems as specific to the patient, in contrast to earlier forms of CBT for bipolar disorder that have focused on preventing future relapse. In a range of other psychiatric disorders, CBT is also designed to work on current problems. Treatment is focused on developing a formulation (a shared understanding) of how thinking styles and behaviors maintain and escalate current symptoms, such as unipolar depression [15, 16], anxiety disorders [17, 18] and eating disorders [19]. In our own research, people diagnosed with bipolar disorders reported that their recovery involved not simply remaining free of relapse, but also regaining a sense of purpose in their lives and facing their longstanding problems [20, 21].

Therefore, the cognitive model [22] that guides TEAMS focuses on understanding what is maintaining people’s current problems, such as anxiety, depression and irritability, and facilitates people regaining control over their lives. The model proposes that people with bipolar disorders experience mood difficulties because they strive to control their mood in extreme ways, which negatively affects their ability to control and achieve their broader goals in life. As such, their mood rarely reaches a stable state and their attempts to pursue their life goals are disrupted. The goal of TEAMS is to help people to develop awareness of the ways in which they manage their moods and develop alternative ways in which to live a more fulfilling and balanced life.

The current study represents the fourth phase in the development and testing of TEAMS. In the first phase, we conducted a series of literature reviews [23–25] and qualitative analyses of personal accounts [21, 26] to inform our treatment model [22]. In the second phase, we tested the model through a range of self-report, observational and experimental studies that provided strong evidence for the central role of extreme appraisals of internal states as a key mechanism in bipolar disorders and mood swings [20, 27, 28]. In the third phase we reported a number of case studies and a case series [29] that demonstrated promising findings regarding acceptability and effect sizes, and so established the rationale for a pilot randomized controlled trial at the fourth stage. At this fourth stage it is important to establish whether there is evidence that TEAMS added to usual care has evidence of clinical effectiveness of usual care. Hence the TEAMS CBT will be compared to usual care. The fifth stage is envisaged to be a multi-center, definitive randomized controlled trial to establish a more precise estimate of the clinical- and cost-effectiveness of the intervention, as well as the generalizability of the results.

## Method

The study was approved by the London Queens-Square Research Ethics Committee (REC reference: 11/LO/1326).

### Objectives

Our objective is to determine whether the TEAMS approach is feasible and acceptable to individuals with bipolar disorder compared with treatment-as-usual and establish an estimate of treatment effect size to inform a larger definitive randomized controlled trial. We aim to assess the feasibility and acceptability of, and information on uptake, service barriers and attrition needed to design a definitive randomized controlled trials. We predict that TEAMS will reduce symptoms of depression, hypomania and anxiety, improve social functioning and promote recovery in comparison to treatment-as-usual at six months post-baseline (post-treatment in the TEAMS wing), and at 12 and 18-months post-baseline. We also aim to evaluate the clinical- and cost-effectiveness of an intervention which has the potential for direct benefits for patients.

### Trial design

A parallel group rater-blind randomized controlled trial comparing treatment-as-usual with up to 16 sessions of TEAMS therapy. Allocation to the two groups will be at a ratio of 1:1 and have an exploratory framework. The design for this study involves a quantitative component (pilot trial) and a qualitative component (interviews with patients and service providers, described later).

### Primary outcome measure

The main objective of the pilot randomized control trial relates to evaluation of recruitment and retention of participants into both arms of the study, as well as adherence to therapy, to determine feasibility and acceptability. We predict that TEAMS will reduce depression in comparison to treatment-as-usual at 6 and 12 months post-randomization, with the Beck Depression Inventory [30] score at six months as the primary outcome measure.

### Secondary outcome measures

The secondary hypotheses are that TEAMS will reduce the severity of hypomanic symptoms and anxiety, change thinking styles responsible for maintaining symptoms which will be targeted by the therapy, improve social functioning and promote recovery compared to treatment-as-usual at post-treatment and follow-up.

Several secondary outcome measures will be used: a Structured Clinical Interview for Diagnosis Longitudinal Interval Follow-up Evaluation (SCID-LIFE) interview for episodes of mania or depression [31], interview measures of manic symptoms (Bech-Rafaelson, [32]) and depression [33], composite internal state scale score (ISS, [34]), anxiety as measured by the Generalised Anxiety Disorder 7 item scale GAD-7 [35], recovery as measured by the Process of Recovery Questionnaire (QPR) [36], cognitive style measured by the Hypomanic Attitudes and Positive Predictions Inventory [37, 38], the EuroQol Health Status Measure (EQ-5D) [39] and session-by-session measures. Raters were trained at the beginning of, and during the study, in interview measures of outcome by an experienced rater who is a consultant psychiatrist (RM), to improve the reliability and validity of the ratings. Participants are paid a fixed amount of expenses for their time (£10 per assessment after baseline) and travel for the follow-up assessment sessions. If any participant withdraws from treatment, we will seek permission to collect outcome data. If a participant misses their assessment, they are contacted a maximum of three times by the rater through the means of contact that they have specified (such as a home or mobile telephone, letter or care coordinator), after which they are coded as missed and re-contacted for the next follow-up point. Self-report measures are collected where interview assessments are not possible.

Prior to each session, the clients complete the ISS and a personalized 10 to 15 item Client version of the Hypomanic Attitudes and Positive Predictions Inventory. (HAPPI) (Client-HAPPI) which assesses their most prominent beliefs about internal states [29]. At the end of each treatment session, clients rate their level of satisfaction with therapy on a Likert scale from 0 (not at all) to 10 (extremely). Adherence is evaluated by the widely used revised version of the Cognitive Therapy Rating Scale (CTS-R) [40], and a brief checklist of adherence specific to the TEAMS model - The TEAMS Treatment Adherence Checklist (T-TAC).

### Think Effectively About Mood Swings therapy

The TEAMS approach involves an individualized treatment plan of CBT based on the integrative cognitive model of bipolar disorder [22]. Specific treatment strategies and therapy techniques employed are determined by an individual formulation using the model. There are several published descriptions of the intervention, as well as a therapy manual in development [14, 22, 29, 41].

### Control condition group

The control condition consists of treatment-as-usual which typically involves regular meetings with a care coordinator, access to a psychiatrist, medication and monitoring of risks that require immediate intervention. Treatments, including other psychological interventions, are not withheld in the treatment-as-usual group but are monitored using a treatment documentation sheet. Following written consent, participants are randomized within two working days.

### Randomization and blinding

–The independent Manchester Academic Health Science Centre Clinical Trials Unit conducts anonymized randomization in a permuted blocks design with block sizes varying randomly between 8 and 12. There is no stratification of the sample, but baseline information may be used in a *post hoc* manner to explore the effects of baseline characteristics. The procedure is as follows. An administrator contacts the clinical trials unit with a password and the participant identification number, and receives an allocation sequence to assign participants to either group. The administrator then directly contacts participants and therapists. Due to the nature of the trial only a single blinding (of raters) is needed. Blindness of raters is ensured using a variety of procedures, including separate offices for therapists and research assistants, briefings to participants prior to assessment and encryption of randomization information. Unblindings are regularly monitored and recorded. Deliberate unblinding would only occur if there was a serious adverse incident such as suicide or violence to another person by a participant.

### Sample size

The primary aim of this study is to evaluate feasibility, therefore a formal power calculation is not appropriate. However, estimates indicate that with 42 participants per group, using a t-test with a two-tailed significance level of 0.05, we will have over 80% power to detect an effect size of 0.8. If the significance level were altered to 15%, 30 participants per group will have 80% power to detect an effect size of 0.6. A previous case series of seven patients generated a pre-post effect size at one-month follow-up on the primary outcome measure of 3.0, and between 0.7 and 1.4 for the secondary measures [29]. The above information makes a feasibility trial of 30 participants per group an appropriate target.

In aiming for 60 participants in total, the target recruitment is 84 participants, allowing a 29% attrition rate by the 12-month post-randomization point. This dropout rate was based on a conservative estimation just above the highest reported rate of attrition found in other published studies of CBT for bipolar disorder (13 [42], 25 [43] and 21% [10]).

### Recruitment

A total of 84 participants aged 16 years or over who meet Diagnostic and Statistical Manual of Mental Disorders Fourth Edition (DSM-IV) criteria for a diagnosis of bipolar disorder will be recruited. Recruitment is taking place across a number of NHS Trusts in and around Greater Manchester. The lead Trust for the research is Greater Manchester West Mental Health NHS Foundation Trust.

Advertisements, including posters and flyers, to promote the study have been distributed in Community Mental Health Teams (CMHT), outpatient clinics, voluntary services (including Manic Depression Fellowship) and GP practices. Local media and websites are used to maximize potential referrals from individuals who are not currently accessing other services. The trial is also supported by the United Kingdom Mental Health Research Network (MHRN) who provide Clinical Studies Officers (CSO) to facilitate recruitment. The research team and CSOs present verbal and written information outlining the study to clinicians, health professionals and voluntary services. Potential participants are offered a participant information sheet containing an overview of the study by a member of their care team, or directly by the research team, if preferred.

### Inclusion and exclusion criteria

Participants meeting all of the following criteria will be included in the study: meet the DSM-IV criteria for bipolar I or II disorder, or bipolar disorder not-otherwise-specified, characterized by a past major depressive episode and DSM-IV hypomania of two days or more; complete a baseline assessment session in an outpatient setting; have a baseline score of at least 15 on the Beck Depression Inventory prior to the study (to ensure presence of significant current distress as targeted in the trial) and be aged 16 years or over.

Participants meeting any of the following criteria will be excluded from the study: have a diagnosis of a non-affective psychotic disorder according to the DSM-IV, currently undergoing mania or a mixed episode according to the DSM-IV, have a primary substance use disorder according to the DSM-IV, have a moderate to severe learning disability, have an organic impairment that accounts for their mental health problem or be non-English speaking (owing to the standardized assessment measures).

### Procedure

**Figure 1:**
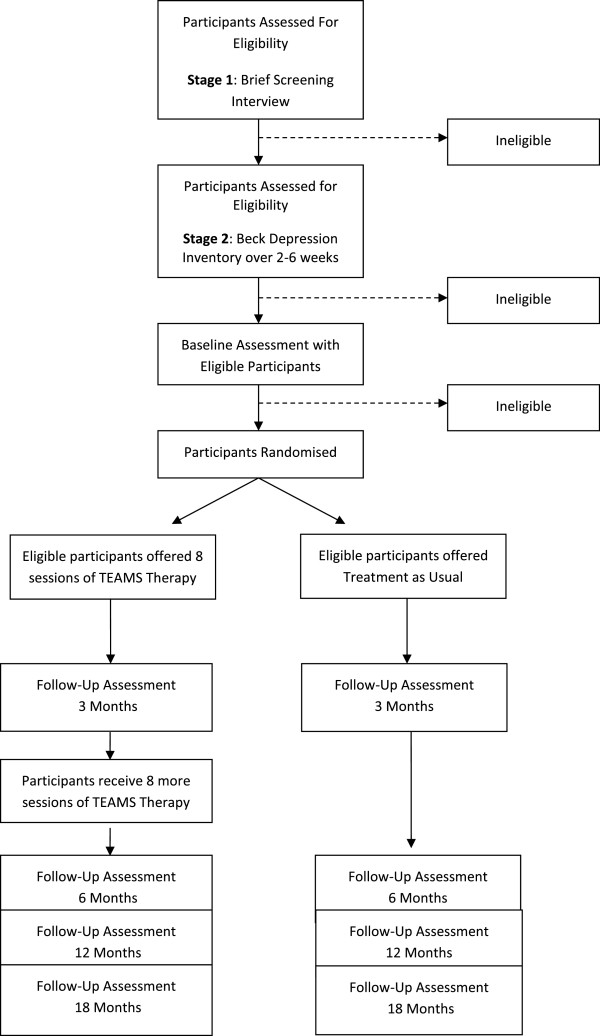
**CONSORT diagram showing design of study.**

Figure 1 (CONSORT diagram) provides a summary of the study procedure. Potential participants are given at least 24 hours to consider the information provided before being contacted by a researcher to discuss the study and ensure that they understand the information provided. For willing participants, the research team asks for written permission to contact their GP and/or other relevant clinician to obtain information that might affect their ability to take part (risk), and to confirm what clinical support will be ongoing during the trial. The research team then conducts a screening assessment to ensure the participant meets the inclusion criteria for the study. This screening process can take place over the telephone or face-to-face (depending on the participant’s preference), and is based on the Brief Screening Interview and two non-consecutive weeks of exceeding the criteria for depressive symptoms (Beck Depression Inventory >15). If inclusion criteria are met at this stage and the potential participant chooses to enter the trial, full written consent will be obtained before the initial baseline assessment.

Full assessments will subsequently be conducted on five occasions: initial baseline, 3 months post-randomization (mid-treatment for the TEAMS group), 6 months post randomization (post-treatment for the TEAMS group), 12 months (6 months post-treatment for the TEAMS group) and 18 months. Those participants who have met the inclusion criteria following baseline assessment are randomized (to treatment-as-usual or TEAMS therapy) and informed of their group allocation by letter. TEAMS treatment usually commences within two weeks of randomization. See Figure 1 (consort diagram) and Table 1.

**Table 1:** **Summary of the measures taken at each assessment session and during therapy**

Assessment	Baseline	3 month follow-up	6 month follow-up	12 month follow-up	18 month follow-up
**Primary outcome measures**					
†Beck Depression Inventory (BDI)	*	*	*	*	*
**Secondary outcome measures**					
Structured Clinical Interview for DSM-IV: Axis I (SCID-I)	*				
Structured Clinical Interview for DSM-IV: Axis II (SCID-II)	*				
Time to bipolar relapse: weekly LIFE scores (SCID life)	*	*	*	*	*
Hamilton Depression Rating Scale (HDRS)	*	*	*	*	*
Bech-Rafaelsen Mania Scale (MAS)	*	*	*	*	*
Use of services interview for economic analysis	*	*	*	*	*
†EuroQol Health Status Measure (EQ-5D)	*	*	*	*	*
†Stephenson Medication Adherence Interview (SMAI)	*	*	*	*	*
†HAPPI-R (Hypomanic Attitudes and Positive Predictions) Inventory)	*	*	*	*	*
†GAD-7 (Generalized Anxiety Disorder - 7 Scale)	*	*	*	*	*
†Internal States Scale (ISS)	*	*	*	*	*
†Questionnaire about the Process of Recovery (QPR)	*	*	*	*	*
**Qualitative measures**					
Patient Experience Interview (PEI; Subgroup)	*				
**Sessional therapy measures**					
^1^Internal States Scale (ISS) graded					
^1^Client HAPPI (tailored and reduced to around 15 items)					
^2^Therapy satisfaction rating					
^3^ T-TAC TEAMS therapist adherence checklist					
^*3*^CTS*-*R *–* cognitive therapy scale *–*revised					

†indicates a self-report measure. All other measures are administered by a researcher as a clinical interview. ^1^Pre-sessional client measures. ^2^Post-session client measures. ^3^Adherence measures. Sessional therapy measures are taken at every assessment.

### Semi-structured interviews and qualitative analyses

Qualitative data forms a significant part of the study and will be utilized to guide the subsequent development of the therapist and client treatment manuals. In this study, we conduct two semi-structured interviews with a subgroup of 12 to 15 randomly selected participants. The interviews were informed on the basis of our previous work on providing TEAMS therapy and through discussions with an experienced service user who is a co-investigator for the trial. The first interview is completed at the baseline and explores participants’ subjective experiences of how they have considered, sought and experienced various forms of treatment in the past, and what they feel an appropriate psychological treatment should involve.

The second interview occurs within six months of treatment ending and examines participants’ subjective experience of receiving the TEAMS intervention. It focuses on what might have been beneficial and what was considered less effective or unhelpful, and eliciting recommendations for future modifications.

Thematic analysis will be used to analyze qualitative data. All interviews are audio recorded and transcribed verbatim. Each transcript will be read and subjected to content analysis to identify themes. Emerging themes from each report will be discussed with the research team to ensure agreement. Data saturation will be regarded as achieved when no new themes emerge (it is likely that this will be achieved with approximately 12 to 15 interviews). Also, a selection of 5 to 10 service providers will be interviewed during the study on their perceptions on their desired outcomes, including referral and retention.

### Data monitoring and management

Hard copies of anonymized data are stored at the NHS site in locked filing cabinets separate from identifiable information (names, addresses and dates of birth). Raters enter data into an electronic database that is stored anonymously with the participant ID only, in a password-protected hard drive. All data entry is checked by a second person and any errors corrected. Only researchers, therapists, study administrators and lead investigators (ST and WM) have access to personal information. Only the lead investigators, trial statistician (GD) and researchers have access to the final dataset.

Adverse and serious adverse events are collected by the research team and reported to the Chief Investigator or their nominated deputy. There is no data monitoring committee because the nature of the intervention is unlikely to result in any adverse events and there are no interim analyses or stopping rules for the trial. Trial conduct may be audited independently of the research team by the sponsor or independently of both the sponsor and the investigators by the funders. In the unlikely event of harm, participants would have access to compensation through the NHS indemnity scheme and through the sponsor of the study.

### Statistical analyses

All primary analyses will be based on an intention-to-treat principle, focusing more on descriptive statistics and confidence interval estimation than statistical significance. Primary and secondary outcomes will be analyzed employing analysis of repeated measures with a mixed-effects model, accounting for the discrete timing of follow-up assessments, and the random censoring introduced by shorter follow-up periods for participants’ recruited towards the end of the trial. The sensitivities of all treatment effect estimates to missing outcome data arising from patient dropout (non-compliance) will be examined and secondary analyses carried out to estimate the Complier-average causal effect (CACE), allowing for missing outcome data, as described in detail [44, 45], again concentrating on confidence interval estimation rather than statistical significance. Basic service data for those refusing randomization will be collected to assess generalizability of the estimated treatment and economic effects. Other than the CACE analyses, there will be no subgroup analysis, interim analysis nor stopping guidelines because there are no circumstances under which the trial would be stopped.

### Cost-effectiveness analysis

The incremental cost-effectiveness ratio of TEAMS therapy will be estimated and cost-effectiveness acceptability analysis conducted, from the perspectives of health and social care providers and patients - the key stakeholders in treatment decisions. Case note review and patient service use questionnaires are being used to collect data about formal and informal service use for each participant to estimate costs for the three months prior to entry to the study and from study entry to end of follow-up. The main measure of health benefit is the quality-adjusted life year, estimated from survival, and the health status of each patient, measured by the EuroQol. Secondary analyses will be used to explore uncertainty due to design decisions and inform the design of a definitive trial.

### Dissemination plans

The findings will be disseminated within peer-reviewed journals authored by the investigators, in addition to presentations at national and international practitioner conferences, and workshops delivered to health professionals as part of training in the TEAMS approach.

## Discussion

This study has been designed to assess the feasibility and acceptability of TEAMS therapy for bipolar disorder and to estimate its effect size ahead of a multi-center randomized controlled trial. TEAMS is based on an integrative cognitive model of bipolar disorder that converges existing theoretical approaches and service-user experiences. Whilst we have established a strong evidence base for several components of the model, the impact of the intervention has been based on case studies and a case series to date.

A key strength of the study is its focus on specific needs and problems as identified by participants. This psychological intervention aims to address the huge impact of current symptoms (including depression, anxiety, irritability and hypomania) and functioning (including life goals around work and social life) that are less emphasized within relapse prevention approaches. The importance of this focus is reflected in the evidence for pervasive subsyndromal symptoms and through the ambivalence reported by many ‘recovered’ people with bipolar disorder as to whether their current wellbeing is satisfactory or complete, given the symptoms and difficulties with functioning they still experience. The importance of focusing treatment on current experiences converges with our model, which suggests that internal states during remission are on a continuum with those in episodes. Therapist and patient develop a shared understanding of the processes that may contribute to the maintenance and escalation of these states and are encouraged to ‘broaden their bandwidth’ of tolerance for such states. This involves understanding, facing, tolerating, enduring and even utilizing internal states (such as fear, anger, excitability, happiness, sadness or tiredness) rather than struggling to suppress or manipulate them. While this therapeutic process often involves accessing past memories of these states and preparing for future situations within which they may arise, the focus is on their experience in the present and how this is managed.

The design of the study incorporates a number of additional strengths, including multiple longitudinal assessment points, which at the developmental stage of a treatment provides important information regarding therapeutic change. Also, researcher-blinded assessments, session-by-session process and adherence measures, and a qualitative analysis of participants’ experiences of treatment as a whole, and TEAMS specifically, should help ensure that the quality of the therapy is carefully considered and that it is modified and enhanced where necessary for future trials and implementations of TEAMS. The model would predict that TEAMS plus treatment-as-usual would lead to reductions in extreme appraisals of internal states compared to treatment-as-usual and that, in turn, this cognitive style post-treatment (six month post-baseline), will be correlated with bipolar symptoms at 12 and 18 months when controlling for other demographic and clinical factors.

There are a number of limitations of the study. The clearest of these is that there is no active psychological intervention of equal intensity in the control group. At this stage of treatment development, it is first important to establish superiority to treatment-as-usual, especially as this patient group is rarely offered such an intervention in routine clinical practice. However, especially given recent developments for bipolar disorder within the Improving Access to Psychological Therapies system, it will be increasingly important to compare TEAMS with an alternative psychological approach. There are many potential active ingredients within TEAMS, including a variety of strategies and techniques, and so this study will not be able to identify the necessary, or most efficient, components. Nevertheless, we will draw upon the TEAMS interview for feedback from participants. Furthermore, participants have consented to recording their sessions for later analysis and so a potential focus of future research is to analyze sessions to identify the components that precede sudden gains in symptoms and functioning.

The study follows up on participants no longer than 18 months post-randomization. Given the scope of the study, this was felt to be appropriate, but longer follow-ups are essential to confirm the efficacy of an intervention for a mental health condition that can involve recurrent relapse. The study is also limited in that it is focused on one regional area, and utilizes only five clinically qualified clinical psychologists and/or CBT therapists who engage in weekly supervision with the developers of the therapy model. Thus, the potential for disseminating TEAMS to other groups, regions and health professionals requires further exploration. TEAMS also has the capacity to be applied to problematic mood swings outside the context of bipolar disorder (such as personality disorder or schizoaffective disorder), yet this was not explored in the current study.

In summary, this protocol is appropriately designed to establish the feasibility, acceptability and initial effect sizes ahead of a multi-center trial with a larger number of participants, therapists and a longer follow-up period. The provision of adherence and process measures and participant interviews will provide additional information and allow convergence with the burgeoning empirical literature regarding the TEAMS model of bipolar disorder.

## Trial status

Participants are being recruited into the trial. Treatment started February 2012 and expected to finish December 2014.

## Abbreviations

CACE: Complier-average causal effect
CBT: Cognitive behaviour therapy
CSO: Clinical studies officers who help to publicize and recruit to studies like this registered with the MHRN
CTS-R: Cognitive Therapy Rating Scale
EQ-5D: EuroQol 5 Dimension
GAD-7: Generalized Anxiety Disorder scale 7 item
HAPPI: Hypomanic Attitudes and Positive Predictions Inventory
ISS: Internal States Scale
MHRN: Mental Health Research Network, a national government funded network to support research studies in mental health registered with the national portfolio in United Kingdom
NHS: National Health Service, United Kingdom
QPR: Process of Recovery Questionnaire
T-TAC: TEAMS Treatment Adherence Checklist
TEAMS: Think Effectively About Mood Swings.
